# Surgical management of complicated inflammatory glaucoma

**DOI:** 10.3205/oc000170

**Published:** 2020-11-10

**Authors:** Glenda Espinosa-Barberi, Francisco José Galván González, David Peláez Viera

**Affiliations:** 1 Institut Català de Retina, Barcelona, Spain; 2 Postgraduate and Doctorate School, University of Las Palmas de Gran Canaria, Spain; 3 Hospital Universitario de Gran Canaria Doctor Negrín, Ophthalmology Department, Las Palmas de Gran Canaria, Spain

**Keywords:** glaucoma, uveitis, inflammatory glaucoma, EXPRESS®, XEN®, Ologen®

## Abstract

**Case report:** We report a case of a 26-year-old woman with a previous history of complicated ulcerative colitis, as well as multiple episodes of recurrent anterior uveitis in control with adalimumab and methotrexate, who develops ocular hypertension refractory to topical treatment. The implant of an EXPRESS^®^ is proposed, but in the immediate post-operative period, the implant causes atalamia and does not achieve the correct control of intraocular pressure. A XEN^®^ stent was implanted. Due to failure, it was decided to remove the stent and to release a subconjunctival fibrosis that had formed at the subconjunctival portion of the XEN^®^, in association with coating by an Ologen^®^ collagen matrix, which led to an improvement of the results.

**Conclusions:** The surgical management of inflammatory glaucoma is complex in young patients with a scar component. The new minimally invasive techniques are effective in cases refractory to topical treatment, whose characteristics prevent the use of conventional ones.

## Introduction

Patients with recurrent episodes of uveitis show an increased risk of developing glaucoma, not only due to intraocular inflammation, but also as a side effect of the corticosteroids used in the treatment [1]. According to studies such as that of Hwang et al. [2], the annual incidence of glaucoma in these patients is approximately 30%, thus being a serious and important complication [3].

Glaucoma are most often caused by anterior uveitis, and most cases are currently attributable to non-infectious entities.

The management of inflammatory glaucoma (IG) is complex. Surgery is often required despite topical treatment and control of the underlying disease, with several postoperative complications. The results of the various treatments and operations are uncertain.

## Case description

A 26-year-old woman undergoing follow-up for acute anterior uveitis (AAU) in the right eye (OR) of 4 years of evolution, in relation to an ulcerative colitis diagnosed in 2005, with a complicated development, underwent total colectomy in 2010, emergency intestinal flange surgery in 2012, and umbilical hemorrhage in 2014. In addition to the surgical interventions, the patient had been treated with mesalazine (5-ASA) orally and rectally; and subsequently with prednisone 1 mg/kg/day, azatriopin (AZA) 150 mg/day and infliximab 5 mg/kg.

In the initial ophthalmologic examination, best corrected visual acuity (BCVA) was 20/20 in both eyes (OU). Anterior segment exploration (BMC) revealed mixed conjunctival hyperemia with + cells in OR. The intraocular pressure (IOP) was 16 mm Hg in OU. Due to the findings, treatment with corticosteroids every hour and topical mydriatics every 8 hours was initiated.

The patient had 4 episodes of AAU, developing posterior synechiae and fibrin membranes, also requiring treatment with subconjunctival injections of triamcinolone, oral prednisone (1 mg/kg/day) and methotrexate (15 mg/week).

Topical and systemic corticosteroids were suspended, using methotrexate (20 mg/week) as maintenance treatment, but a decrease in BCVA was observed in the patient’s OR (20/30). Funduscopic examination showed a cystic macular edema (CME) of aproximately 400 microns, so treatment with acetazolamide 375 mg/day was initated.

At 6 months, the BCVA decreased (20/60), and the development of a posterior subcapsular cataract was observed.

**Figure 1:**
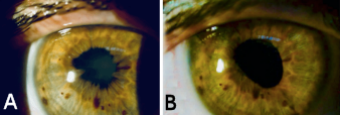
Exploration of the anterior segment (A) before and (B) after cataract and synechiotomy surgery

Adalimumab treatment (40 mg/2 weeks) was initiated to control intraocular inflammation. The patient presented episodes of increased IOP in the OR, ranging between 26–36 mm Hg, with no signs of intraocular inflammation. An open angle was observed in gonioscopy (Shaffer grade III), with some previous peripheral synechiae, so treatment with brimonidine, timolol, and brinzolamide was added every 12 hours. Due to inactivity, phacoemulsification was performed with iris retractors and intraocular lens implants in the capsular bag in the OR after synechiotomy (Figure 1 (Fig. 1)).

After surgery, the BCVA improved (20/30), but adequate control of the IOP was not achieved, and signs of glaucoma progression in the OR were observed by spectral domain optical coherence tomography (SD-OCT), as well as in the visual field (VF): 24.2 (MD=–1.94 dB).

A protected sclerostomy and implant of an EXPRESS^®^ (Optonol LTD, Kansas City, MO) was performed in 2015.

In the early postoperative period, the IOP increased to 38 mm Hg, decreasing to 33 mm Hg after massage, so topical treatment with combination of timolol and bimatoprost was initiated every 24 hours. Subconjunctival 5-fluoracil was injected in two sessions.

Although the IOP was maintained in a range between 16 and 21 mm Hg, there were signs of progression. Consequently, suspecting IOP peaks, the implantation of a XEN^®^ stent was planned (XEN^®^ Gel Stent, AqueSys, Wisconsin, USA) in 2017.

At two weeks after surgery, the pressure increased. In gonioscopy as well as in BMC examination, the XEN^®^ stent was well positioned, with no signs of intraocular inflammation. However, the bleb was flat and did not rise despite the massage. On the other hand, the EXPRESS^®^ was not functioning and superficial, with a high risk of extrusion.

**Figure 2:**
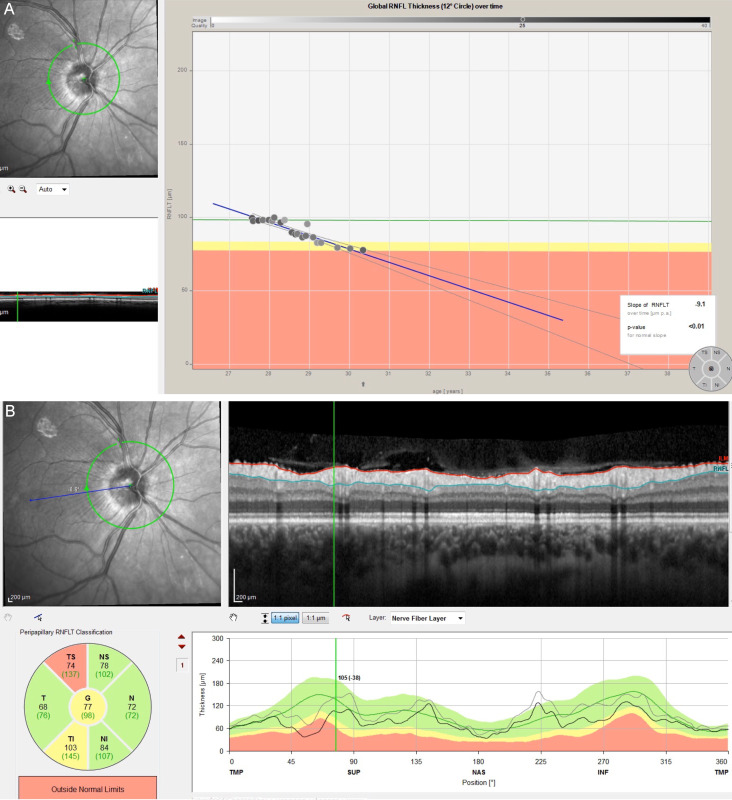
(A) Progression analysis using optical coherence tomography from the first raise of the intraocular pressure (2014); (B) Spectral domain optical coherence tomography with analysis of nerve fiber layers at the level of the optic nerve in which there is involvement of moderate-severe temporal sectors

Optic nerve SD-OCT showed loss of retinal nerve fiber layers thickness. In the 24.2 VF, an increase in nasal scotoma (MD=–5.42 dB) was shown (Figure 2 (Fig. 2)).

**Figure 3:**
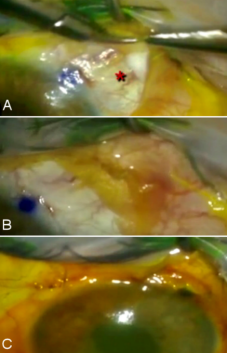
(A, B) Release of fibrous adhesions at the distal end of the XEN^®^ stent and placement of Ologen^®^ collagen matrix surrounding the subconjunctival tubular lumen; (C) Final appearance of the anterior chamber and fluorescein staining to corroborate the absence of Seidel test

We decided to remove the EXPRESS^®^ and to release a fibrosis that had formed in the outer third of the XEN^®^ (two months after implantation), in combination with the implantation of an Ologen^®^ collagen matrix implant as a sandwich, surrounding the subconjunctival portion (Figure 3 (Fig. 3)).

**Figure 4:**
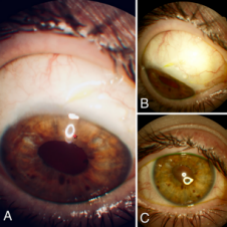
Current appearance of the anterior chamber (17 months after surgery) in which the XEN^®^ implant (A) is well positioned, formation of upper nasal bleb (B) and absence of inflammatory activity (C)

Currently (17 months later), the patient has a BCVA of 20/25 in the right eye (OR) and 20/20 in the left eye (OS). In BMC, there are no signs of activity, and the IOP is at levels between 10–14 mm Hg in OU (without topical or systemic hypotensive treatment) (Figure 4 (Fig. 4)).

## Discussion

The mechanisms that determine the increase in IOP in patients with chronic uveitis are complex and multifactorial [4]. In our patient, we were able to appreciate a complicated base disease that was difficult to control, which determined the inflammatory course of ocular pathology. Various parameters indicative of unfavorable evolution were observed, which in turn were predictors of the need for surgical treatment, such as corticoresistance, chronic and recurrent inflammation, and increased resistance of the trabecular mesh due to synechial and cyclic membrane formation.

In the clinical case, the first surgery to be performed was the extraction of the cataract and the release of the synechia, since this could improve vision, reduce IOP, and increase the space in the anterior chamber to avoid pupillary blockage (a frequent complication in inflammatory glaucoma caused by the presence of primary synechiae that generate gradual closure) [5].

Due to the uncontrolled pressure and the presence of signs of progression (deterioration of the nerve fiber layer in the OCT and worsening at the level of the VF), we considered the performance of a filtering surgery in a second step. In these cases, trabeculectomy in association with antimetabolite agents has shown good short-term results. However, the high risk of hypotonia, and the significant inflammatory reaction – and therefore the post-operative surgical failure – we were faced with when manipulating the iris, caused us to look for other alternatives. We ruled out the Ahmed valve implant and the Baerveldt implant because the young age of the patient required an option with long-term survival, so we chose the EXPRESS^®^ implant [6], [7].

Protected sclerostomy with an EXPRESS^®^ implant is a safe, effective procedure with a short learning curve. It is an excellent alternative in patients with open-angle refractory glaucoma and with anatomical and functional contraindications for conventional procedures as in our case [6], [7]. Among this procedure’s complications, it is worth highlighting the severe hypotonia in the immediate postoperative period [7], an event that we experienced, but which resolved after strengthening the scleral mat. In the short term, the device became non-functioning, so another filtering surgery was required. We opted for a minimally invasive alternative and took advantage of the development of new techniques such as the XEN^®^ stent.

Because there is little manipulation of conjunctival tissue with the stent, the risk of complications of the inflammatory type is minimal. Studies such as that of Schlenker et al. [8] compare XEN^®^ with trabeculectomy. They find that there are no significant differences with regard to safety, and that the implant has no serious complications. In our case, the stent was losing its effectiveness due to the creation of a fibrin capsule that occluded the distal tubular lumen. This was one of the main causes of failure according to various studies, such as the review by Chaudhary et al. [9]. Since the implant was well positioned at the level of the anterior chamber and the problem was at the distal level, we proceeded to review the blister, released this end of the XEN^®^, and used the Ologen^®^ collagen matrix. We chose this approach instead of performing a conventional needling with 5-fluorouracil, because it has been shown that when the Ologen^®^ collagen matrix is combined with glaucoma surgeries, the results in terms of efficacy are better than with the use of antimetabolites, and because it achieves a significant improvement in cases of post-operatory hypotonia [10].

This case demonstrates the difficult surgical management of IG. It is usually young patients who experience a large inflammatory and scar component, and consequently show a high rate of surgical failure and complications. The follow-up must be close, and control of the base disease is important in order to reduce activity outbreaks. The Ologen^®^ collagen matrix is a good alternative to the needling in cases where XEN^®^ obstruction is suspected due to non-control of the IOP and/or where the ampoule is not the desired one. However, more extensive studies are required in this regard in order to determine its effectiveness.

## Notes

### Competing interests

The authors declare that they have no competing interests.
